# NONTRADITIONAL VIEWS? HOW SIBLINGS MATTER FOR PERCEIVED PARENTAL CARE RESPONSIBILITY IN JAPAN

**DOI:** 10.1093/geroni/igz038.1817

**Published:** 2019-11-08

**Authors:** Ryo Hirayama, Tomoko Wakui

**Affiliations:** Tokyo Metropolitan Institute of Gerontology, Tokyo, Japan

## Abstract

Our aim in this study was to explore whether and how siblings’ marital and work status influence Japanese adult children’s perceived responsibility for parental care. Within traditional familial institutions in Japan, married sons were expected to assume parental care responsibility. At the same time, such care arrangements built on gendered division of labor; sons served as family breadwinners, and their wives cared for their parents-in-law while out of the paid labor force. Yet, because of sociodemographic shifts such as a greater percentage of unmarried persons and a growing number of women who seek to maintain their job, it has been increasingly unclear which adult children can and should assume the role of parental caregiver. Using online survey data from 989 Japanese adult children who were all employees with no parental care experiences ever, we sought to clarify the influences of siblings’ circumstances on whether these children anticipated assuming responsibility for conducting different care tasks for their parents. In doing so, we focused on how siblings’ gender and work and marital status might combine to affect adult children’s anticipation of parental care responsibility. A series of logistic regression analyses revealed that having a married brother made it less likely for adult daughters to anticipate assuming responsibility for conducting typical care tasks (e.g., ADL assistance) whereas for adult sons, having a single sister declined such anticipation. We discuss our findings in terms of how traditional familial institutions still impinge on Japanese adult children’s views of parental care responsibility.

